# Regulation of 8-Hydroxydaidzein in IRF3-Mediated Gene Expression in LPS-Stimulated Murine Macrophages

**DOI:** 10.3390/biom10020238

**Published:** 2020-02-04

**Authors:** Nur Aziz, Young-Gyu Kang, Yong-Jin Kim, Won-Seok Park, Deok Jeong, Jongsung Lee, Donghyun Kim, Jae Youl Cho

**Affiliations:** 1 Department of Integrative Biotechnology, Sungkyunkwan University, Suwon 16419, Korea; nuraziz@skku.edu (N.A.); jd279601@gmail.com (D.J.); 2 Basic Research & Innovation Division, R&D Center, AmorePacific Corporation, Yongin 17074, Korea; kangyg82@amorepacific.com (Y.-G.K.); jaykim@amorepacific.com (Y.-J.K.); wspark@amorepacific.com (W.-S.P.)

**Keywords:** 8-Hydroxydaidzein, IRF-3, TRAF3, IKKε, inflammation

## Abstract

Cytokines and chemokines are transcriptionally regulated by inflammatory transcription factors such as nuclear factor-κB (NF-κB), activator protein-1 (AP-1), and interferon regulatory factor (IRF)-3. A daidzein derivative compound, 8-hydroxydaidzein (8-HD), isolated from soy products, has recently gained attention due to various pharmacological benefits, including anti-inflammatory activities. However, regulation of the inflammatory signaling mechanism for 8-HD is still poorly understood, particularly with respect to the IRF-3 signaling pathway. In this study, we explored the molecular mechanism of 8-HD in regulating inflammatory processes, with a focus on the IRF-3 signaling pathway using a lipopolysaccharide (LPS) and polyinosinic:polycytidylic acid [Poly (I:C)] stimulated murine macrophage cell line (RAW264.7). The 8-HD downregulated the mRNA expression level of IRF-3-dependent genes by inhibiting phosphorylation of the IRF-3 transcription factor. The inhibitory mechanism of 8-HD in the IRF-3 signaling pathway was shown to inhibit the kinase activity of IKKε to phosphorylate IRF-3. This compound can also interfere with the TRIF-mediated complex formation composed of TRAF3, TANK, and IKKε leading to downregulation of AKT phosphorylation and reduction of IRF-3 activation, resulted in inhibition of IRF-3-dependent expression of genes including *IFN-β*, C-X-C motif chemokine 10 (*CXCL10*), and interferon-induced protein with tetratricopeptide repeats 1 (IFIT1). Therefore, these results strongly suggest that 8-HD can act as a promising compound with the regulatory function of IRF-3-mediated inflammatory responses.

## 1. Introduction

Immune cells detect pathogens by the engagement of pathogen-associated molecular patterns (PAMPs) into pattern recognition receptors (PRRs). Subsequently, innate immune signaling cascades are triggered, which lead to the activation of various transcription factors such as NF-κB, AP-1, and interferon regulatory factor (IRF) [1,2]. Following activation of any of these transcription factors, innate immune cells then begin to produce various types of chemokines and cytokines that are critical for eliminating the invading pathogen [3]. IRF-3 is a transcription factor known to be involved in a variety of cellular responses, specifically, innate immune responses to bacterial and viral infections [4,5]. Particularly, initial sensing by different types of PRRs, such as Toll-like receptors (TLRs), RIG-I-like receptors (RLRs), and cytosolic DNA sensors, e.g., cyclic GMP-AMP synthase (cGAS) and stimulators of interferon genes (STING), has been reported to induce activation of IRF-3 [6]. Signaling cascades then converge to activate IRF-3, which is mediated by its phosphorylation on Ser396. This event allows IRF-3 dimerization and translocation to the nucleus, followed by transcription of IRF-3-dependent genes such as type I interferons, *CXCL10*, and chemokine (C-C motif) ligand 5 (*CCL5*) [7]. These cytokines and chemokines then act as molecular effectors for pathogen removal of molecular mediators to activate immune cells and promote adaptive immunity. 

Inflammatory processes are indeed a defense mechanism as part of an advanced ability of the body to maintain tissue homeostasis against harmful stimuli including pathogens as well as tissue injury, stress, and malfunction. In addition, inflammation, particularly chronic inflammation, is widely known to be a common pathogenesis of many chronic diseases such as chronic hepatitis, hepatocirrhosis, gastric ulcer, cardiovascular and bowel diseases, diabetes, arthritis, and cancer [1,8,9]. For instance, when acute inflammation fails to eliminate the source of an injury, the resolution of the inflammation cannot be achieved. As a result, progression from acute inflammation to persistent/chronic inflammation occurs. Since chronic inflammation has been implicated in various serious diseases such as cancer, a growing realization of the crucial role of inflammatory processes in the pathogenesis of chronic diseases makes it possible to develop a new generation of drugs to prevent these types of diseases by treating inflammatory processes [10]. In other words, developing an agent that can regulate the inflammatory processes could help prevent such serious diseases [11].

**Figure 1:**
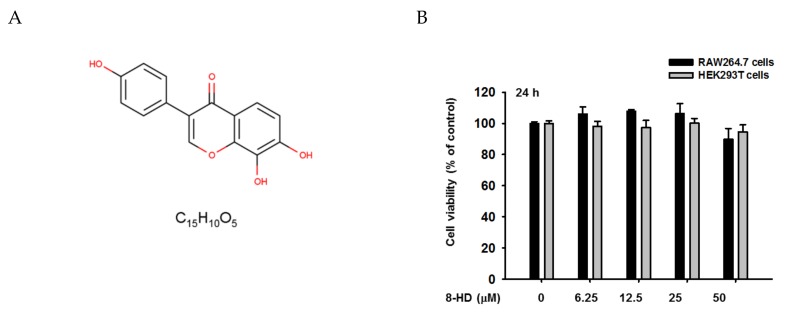
Chemical structure and viability profiles of 8-hydroxydaedzein (8-HD). (**A**) Chemical structure and a molecular formula of 8-HD. (**B**) RAW264.7 and HEK293T cells (10^5^ cells/well) were treated with 8-HD at the indicated doses for 24 h, and cell viability was analyzed by a 3-(4,5-dimethylthiazol,2-yl)-2,5-diphenyltetrazolium bromide (atetrazole) (MTT) assay.

Isoflavones, a subclass of flavonoids, are mainly found in soybeans, soy foods, and legumes [12]. Daidzein and genistein are the most common isoflavones found in soybeans [13]. It has been reported, in view of structure-activity relationships, that the number and positions of functional groups in the chemical structures of isoflavones can dramatically affect their functions [13]. 8-hydroxydaidzein (8-HD) (Figure 1) is a daidzein derivative with increasing evidence in support of its bioactivities. Several bioactivities of 8-HD have been reported with regard to anti-cancer-related activity such as anti-proliferative and anti-mutagenic activity, anti-melanogenic activities such as an irreversible tyrosinase inhibitor, and other bioactivities such as aldose reductase inhibitor and depigmenting activities [13,14]. In regard to inflammation, 8-HD has been recently reported to exert anti-inflammatory activities via activation of Nrf2-antioxidant, inhibition of AKT phosphorylation in NF-κB-signaling pathways, and suppression of TAK1 activation followed by decreasing phosphorylation levels of downstream signaling molecules in AP-1 activation pathways, which are mostly triggered by bacterial and fungal infections [15,16]. Although the anti-inflammatory activities of this compound have been described, there have been no reports regarding the regulatory activities of 8-HD with respect to the IRF-3 signaling pathway which is important in virus- or virus/bacterium-induced inflammatory responses [17]. In this study, we investigated the role of this compound in the regulation of the IRF-3 signaling pathway in the LPS- and polyinosinic:polycytidylic acid (Poly (I:C))-stimulated murine macrophage cell line RAW264.7 and revealed underlying mechanisms of its regulation.

## 2. Materials and Methods 

### 2.1. Materials

8-Hydroxydaidzein (8-HD) was purchased from INDOFINE Chemical Company, Inc. (Hillsborough, NJ, USA). Sodium dodecyl sulfate (SDS), 3-(4,5-dimethylthiazol,2-yl)-2,5-diphenyltetrazolium bromide (atetrazole) (MTT), polyethylenimine (PEI), dimethyl sulfoxide (DMSO), polyinosinic:polycytidylic acid (Poly I:C), and lipopolysaccharide (LPS, *E. coli* 0111:B4) were purchased from Sigma-Aldrich Co. (St. Louis, MO, USA). Adenosine 5′-triphosphate (ATP) was purchased from New England Biolabs, Inc. (Ipswich, MA, USA). Roswell Park Memorial Institute (RPMI) 1640, Dulbecco’s modified Eagle’s medium (DMEM), penicillin-streptomycin, and trypsin were purchased from HyClone (Logan, UT, USA). Fetal bovine serum (FBS) was purchased from Biotechnics Research, Inc. (Irvine, CA, USA). TRIzol reagent was purchased from MRCgene (Cincinnati, OH, USA). Phosphate-buffered saline (PBS) was purchased from Capricorn Scientific GmbH (Ebsdorfergrund, Germany). Phosphospecific or total-protein antibodies raised against IRF-3, β-actin, TBK1, IKKε, AKT, AKT1, AKT2, lamin A/C, Flag, and HA were purchased from Cell Signaling Technology (Beverly, MA, USA). Anti-TRAF3 antibody was obtained from Santa Cruz Biotechnology, Inc. (Dallas, TX, USA). *IFN-β* primers used for the semiquantitative reverse transcriptase (RT)-polymerase chain reaction were purchased from Bioneer Corp. (Daejeon, Korea). Additional primers used in this study were purchased from Macrogen Inc. (Seoul, Korea). PCRBIO HS Taq PreMix and qPCRBIO SyGreen Blue Mix Lo-ROX for PCR were purchased from PCR Biosystems Ltd. (London, UK). Constructs for signaling molecules such as Flag-TBK1-WT, HA-AKT1, and HA-AKT2 were used as reported previously [18,19]. pcDNA3-IKKε-Flag was a gift from Tom Maniatis (Addgene plasmid #26201, http://n2t.net/addgene:26201 RRID:Addgene_26201) [20]. Flag-TBK1-ΔCC (TBK1 plasmid DNA mutant of the CC domain) was constructed by a standard cloning method. All constructs were confirmed by automated DNA sequencing. RAW264.7 cells (ATCC number TIB-71) and HEK293T cells (ATCC number CRL-1573) were purchased from the American Type Culture Collection (ATCC) (Rockville, MD, USA).

### 2.2. Cell Culture and Compound Preparation

A BALB/c-derived murine macrophage cell line (RAW264.7) was cultured in RPMI 1640 media supplemented with 10% heat-inactivated FBS, 100 U/mL of penicillin, 100 μg/mL of streptomycin, and 2 mM l-glutamine. A human embryonic kidney cell line (HEK293T) was cultured in DMEM media supplemented with 5% heat-inactivated FBS, 100 U/mL of penicillin, 100 μg/mL of streptomycin, and 2 mM l-glutamine. Both cell lines were grown at 37 °C under 5% CO_2_ in a humidified incubator. The stock solution of 8-HD was prepared by dissolving the 8-HD powder in 100% DMSO in a microcentrifuge tube. The use of DMSO treatment in the following study is in the same concentration as DMSO content in the diluted compound (8-HD).

### 2.3. Cell Viability Assay

The cytotoxic effect of 8-HD on tested cells (RAW264.7 and HEK293T cells) was evaluated by conventional MTT assay as reported previously [21]. For instance, cells (10^5^ cells/well) were plated in 96-well plates and incubated overnight, followed by 8-HD (0, 6.25, 12.5, 25, and 50 μM) treatment for 24 h. Next, 10 μL of MTT solution (10 mg/mL in PBS pH 7.4) was added to the cell culture for 3 h at 37 °C. The reaction then stopped by adding 100 μL stop solution (15% sodium dodecyl sulfate), followed by incubation for 8 h at 37 °C. The absorbance was then measured at 570 nm using a Synergy HT Multi-Mode Microplate Reader (BioTek Instruments GmbH, Bad Friedrichshall, Germany).

### 2.4. mRNA Expression Analysis by Semiquantitative Reverse Transcriptase (RT)-Polymerase Chain Reaction (PCR) and Quantitative Real-Time PCR (qPCR)

RAW264.7 cells (10^6^ cells/well) were pre-incubated overnight, followed by incubation with 8-HD (0, 12.5, 25, and 50 μM) for 30 min and additional incubation with LPS (1 μg/mL) for 6 h or poly I:C for 18 h. Isolation of total RNA from these cells was performed using TRIzol reagent according to the manufacturer’s instructions. For this, 1 μg of total RNA was used for cDNA synthesis using a cDNA synthesis kit (Thermo Fisher Scientific, Waltham, MA, USA) according to the manufacturer’s instructions. Analysis of mRNA expression by semiquantitative RT-PCR and qPCR was conducted as previously described [22,23]. The primer sequences used in this study are listed in Table 1. 

### 2.5. siRNA Transfection

RAW264.7 cells (5 × 10^5^ cell/mL) were pre-incubated overnight, followed by transfection with IKKε-small interfering RNA (siRNA) 60 nM for 48 h by using Lipofectamine RNAiMAX (Thermo Fisher Scientific, Waltham, MA, USA) according to the manufacturer’s instructions. The oligonucleotides sequences of siRNA targeting IKKε as following: sense: 5′-GAUUCAUGAGGAUAACAAATT-3′ and antisense: 5′-UUUGUUAUCCUCAUGAAUCTG-3′. After 48 h siRNA transfection, the cells were then treated with LPS (1 μg/mL) for 30 min. The knockdown level was confirmed by using western blot analysis. For mRNA analysis, the cells were treated with LPS (1 μg/mL) for 6 h.

### 2.6. Preparation of Whole-Cell Lysates, Nuclear Fraction, and Immunoblotting

HEK293T cells (5 × 10^5^ cell/mL) were pre-incubated overnight, followed by transfection with plasmid DNA such as that of Flag-TBK1-WT, Flag-TBK1-ΔCC, HA-AKT1, HA-AKT2, and Flag-IKKε or an empty vector using PEI for 24 h. These cells were then treated with 8-HD (50 μM) for an additional 24 h without changing the media. RAW264.7 cells (2.5 × 10^6^ cell/mL) were pre-incubated overnight, followed by pre-treatment with 8-HD (50 μM) for 30 min, then the cells were incubated with LPS (1 μg/mL) for 30, 60, 90, and 120 min. Both cell types were harvested at the end of the incubation as indicated, and whole-cell lysates were prepared as described previously [24]. For nuclear fraction preparation, RAW264.7 cells (5 × 10^6^ cell/mL) were pre-incubated overnight, followed by pre-treatment with 8-HD (50 μM) for 30 min and an additional 30 min incubation with LPS (1 μg/mL). The nuclear fraction was prepared as described previously [25]. Immunoblotting of both nuclear fraction and whole-cell lysate were performed as previously described [24,26]. β-actin and lamin A/C were used as a loading control for the whole cell lysate and nuclear fraction, respectively.

### 2.7. Immunoprecipitation-Kinase Assay

For the in-vitro kinase assay in Figure 4A [27], we collected an enzyme to be used for immunoprecipitation of p-IKKε from pre-incubated RAW264.7 cells (2.5 × 10^7^ cell/mL) treated with or without LPS (1 μg/mL) for 30 min as indicated. In parallel, we also collected a substrate by immunoprecipitation of IRF-3 from untreated RAW264.7 cells (2.5 × 10^7^ cell/mL). Immunoprecipitates of both enzyme and substrate were prepared in kinase buffer containing 25 mM Tris pH 7.5, 5 mM β-glycerophosphate, 2 mM DTT, 0.1 mM Na_3_VO_4_, and 10 mM MgCl_2_. The in-vitro kinase assay was performed by incubation of enzyme, substrate, and ATP (400 μM) in the presence or absence of 8-HD (50 μM) for 1 h at 37 °C. The reaction was terminated by adding 1× sample buffer, and the solution was denatured at 95 °C for 5 min. These mixtures were then subjected to immunoblotting analysis. 

For the immunoprecipitation assay in Figure 4B, pre-incubated RAW264.7 cells (2.5 × 10^7^ cell/mL) were treated with or without 8-HD (50 μM) for 30 min, followed by an additional incubation with or without LPS (1 μg/mL) for 30 min as indicated. Cell lysates for immunoprecipitation were prepared in immunoprecipitation buffer containing 50 mM Tris-HCl pH 7.5, 20 mM NaF, 25 mM β-glycerophosphate pH 7.5, 120 mM NaCl, 2% NP-40, 2 μg/mL leupeptin, 2 μg/mL aprotinin, 2 μg/mL pepstatin A, 0.1 mM Na_3_VO_4_, 1 mM benzamide, 0.1 mM PMSF, and 1.6 mM pervanadate. Cell lysates containing equal amounts of protein (1000 μg/sample) were collected and incubated with a specific antibody overnight at 4 °C with constant rotation (25 rpm). The complex antigen-antibodies were then captured on 50 μL of protein A or G-coupled Sepharose beads (50% *v*/*v*) and incubated for 4 h at 4 °C with constant rotation (25 rpm). Sepharose beads were then collected by pulsing in microcentrifuge tubes at 10,000 rpm for 1 min. Pellets were washed 3 times with immunoprecipitation buffer. Protein elution was performed by adding 50 μL of 2× sample buffer and was denatured at 95 °C for 5 min. These mixtures were then subjected to immunoblotting analysis.

### 2.8. Cellular Thermal Shift Assay (CETSA)

RAW264.7 cells (2.5 × 10^7^ cell/mL) were seeded in 10 cm plates. After overnight growth, the cells were treated with 8-HD (50 μM) or DMSO for 30 min, followed by an additional incubation with LPS (1 μg/mL) for 30 min. The cells were washed with PBS and collected in PBS. After cells centrifugation (1000 g for 5 min), PBS were removed, and cells pellets was resuspended with 1 mL PBS containing 20 mM Tris-HCl pH 7.4, 100 mM NaCl, 5 mM EDTA, 2 mM PMSF, 10 ng/mL leupeptin, and 10 μg/mL aprotinin. Cells were then distributed into PCR tubes, with 100 μL of cell suspension in each tube. Each tube was marked with a designated heat temperature followed by heat treatment at the designated temperature using PCR machines. Heat treatment was set for 3 min at each designated temperature following 3 min incubation at 25 °C, and immediately snap-frozen in liquid nitrogen. The cells were further lysed over 4 freeze-thaw cycles by alternating liquid nitrogen and a heating block set at 25 °C. The lysate was then transferred to Eppendorf and centrifuged at 15,000 g for 25 min at 4 °C. The supernatant was added with 1 × sample buffer and denatured at 95 °C for 5 min, these mixtures were then subjected to immunoblotting analysis.

### 2.9. Statistical Analysis

All data in this study are presented as the mean ± standard deviation of at least three independent replicate experiments. Statistical comparisons were analyzed using either analysis of variance (ANOVA)/Scheffe’s post hoc test or the nonparametric Kruskal-Wallis/Mann-Whitney test. A value of p<0.05 was considered a statistically significant difference. All statistical analysis was performed using SPSS software (IBM Corporation, Armonk, NY, USA). 

## 3. Results

### 3.1. 8-HD Downregulates IRF-3-Dependent Genes in LPS-Stimulated RAW264.7 Cells

**Figure 2:**
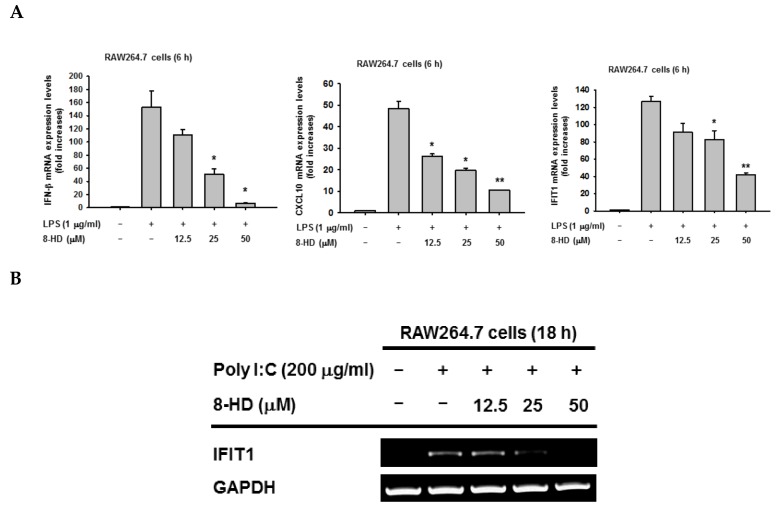
8-HD downregulates mRNA expression of IRF-3- dependent genes. RAW264.7 cells (10^6^ cells/mL) were pre-treated with 8-HD for 30 min, followed by incubation with LPS (1 μg/mL) for 6 h or Poly I:C (200 μg/mL) for 18 h. mRNA expression levels of IRF-3-dependent genes (*IFN-β*, *CXCL10,* and IFIT1) were determined by real-time PCR (**A**) or semiquantitative reverse transcriptase PCR (RT-PCR) (**B**). *GAPDH* was used as a loading control. All data are expressed as mean ± SD. ∗ *p* < 0.05 and ∗∗ *p* < 0.01 compared to the control group (LPS alone).

To determine whether 8-HD can regulate the IRF-3 signaling pathway, we investigated whether 8-HD could alter the mRNA expression of IRF-3-dependent genes such as *IFN-β*, *CXCL10,* and *IFIT1*. Using real-time PCR (Figure 2A) and semi-quantitative RT-PCR (Figure 2B), the mRNA expression level of IRF-3-dependent genes was analyzed in LPS- and poly (I:C)-stimulated RAW264.7 cells pre-treated with 8-HD (12.5, 25, and 50 μM) for 30 min. The results of both methods indicated that 8-HD was able to dose-dependently downregulate the mRNA of *IFN-β*, *CXCL10,* and *IFIT1* genes. To further confirm that these effects are not due to cytotoxicity of 8-HD, we performed the 3-(4,5-dimethylthiazol,2-yl)-2,5-diphenyltetrazolium bromide (atetrazole) (MTT) assay in tested cells. As shown in Figure 1B, 8-HD did not exhibit any cytotoxic effect, observed as no significant decrease in cell viability at concentrations up to 50 μM in RAW264.7 cells. These results indicated that 8-HD inhibits mRNA expression of IRF-3-dependent genes without showing any cytotoxicity, providing evidence that 8-HD might regulate the IRF-3 signaling pathway. 

### 3.2. 8-HD Inhibits Phosphorylation IRF-3 and Nuclear Translocation of IRF-3

**Figure 3:**
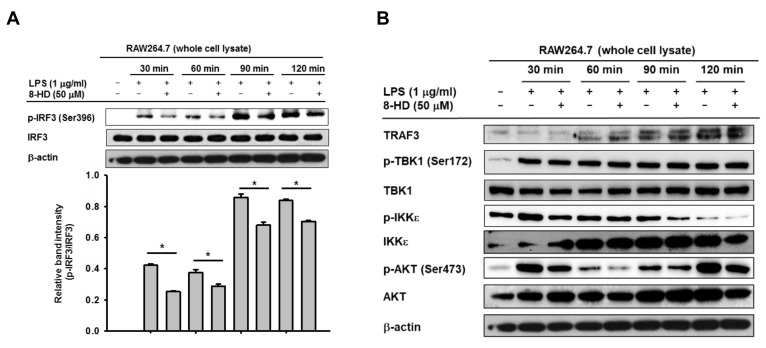
8-HD inhibits activation of IRF-3 by regulating upstream enzymes. RAW264.7 cells (2 × 10^6^ cells/mL) were pre-treated with 8-HD (50 μM) for 30 min, followed by incubation with LPS (1 μg/mL) for the indicated time periods. (**A**) The effect of 8-HD on activation of IRF-3 in LPS-induced RAW264.7 cells. Phospho- and total levels of IRF-3 in total cell lysates were determined by immunoblotting (**B**) The effect of 8-HD on upstream enzymes of IRF-3 signaling (p-AKT, AKT, p-IKKε, TBK1, p-TBK1, and TRAF3) in the whole-cell lysates were determined by immunoblotting. β-Actin was used as a loading control for whole-cell lysates. ∗ *p* < 0.05 compared to the control group (LPS alone).

As 8-HD was shown to downregulate mRNA expression of IRF-3-dependent genes in LPS-stimulated RAW264.7 cells, we confirmed that 8-HD can indeed regulate the IRF-3 signaling pathway by analyzing IRF-3 activation at protein levels. A hallmark of IRF-3 activation is mediated by a phosphorylation event at Ser396, followed by dimerization and translocation of the IRF-3 dimer to the nucleus. We examined the level of IRF-3 phosphorylation at Ser396 in whole-cell lysate from LPS-stimulated RAW264.7 cells pre-treated with or without 8-HD (50 μM) at different time points after LPS-stimulation by immunoblotting. As shown in Figure 3A, pre-treatment with 8-HD (50 μM) decreased the phosphorylation level of IRF-3 at Ser396. In similar conditions, we evaluated the level of IRF-3 translocated into the nucleus by using nuclear fractionation methods as previously reported [23]. Interestingly, Figure S2 indicated that 8-HD (50 μM) was also able to reduce IRF-3 translocation into the nucleus.

### 3.3. 8-HD Alters Upstream Signaling Enzymes of the IRF-3 Signaling Pathway

We next investigated the effect of 8-HD on upstream signaling enzymes responsible for IRF-3 activation. Using the whole-cell lysate used for analyzing p-IRF-3 level, we also determined the protein level of upstream enzymes in the IRF-3 signaling pathway, including protein kinase B (AKT), inhibitor of nuclear factor kappa-B kinase subunit epsilon (IKKε), TNF receptor-associated factor 3 (TRAF3), TRAF family member-associated NF-κB activator (TANK), and TANK binding kinase 1 (TBK1). The immunoblotting results indicated that 8-HD decreased the phosphorylation of AKT and IKKε but had no effect on that of TBK1 (Figure 3B). These results showed that 8-HD might inhibit IRF-3 activation due to inhibition of IKKε and AKT. Precisely, the molecularly targeted inhibition of 8-HD might specifically be caused by inhibition of IKKε phosphorylation, since AKT is downstream of TBK1/IKKε, thus also reducing AKT phosphorylation [28,29]. 

To confirm this hypothesis, we examined the autophosphorylation levels of TBK1, AKT, and IKKε after treatment with 8-HD (50 μM) by overexpression of these molecules in HEK293T cells. An MTT assay demonstrated that 8-HD had no significant toxicity in HEK293T cells (Figure 1B). We transfected TBK1- WT and TBK1-ΔCC, a domain deletion mutant of TBK1 known to maintain TBK1 kinase activity [30], for 24 h, followed by 8-HD (50 μM) treatment for 24 h without changing the media in HEK293T cells. In another trial using similar conditions, we transfected AKT1, AKT2, and IKKε into HEK293T cells. Whole-cell lysates were prepared from these cells to be analyzed by immunoblotting. The result indicated that 8-HD (50 μM) had no effect on autophosphorylation of TBK1 (Figure S1A) or AKT (Figure S1B). These results implied that 8-HD had no effect on phosphorylation of TBK1 and had an effect on AKT phosphorylation mediated by inhibition on IKKε rather than by direct inhibition on AKT, shown by lack of inhibition of AKT autophosphorylation with the 8-HD treatment condition.

### 3.4. 8-HD Inhibits the Kinase Activity of IKKε to Phosphorylates IRF-3

**Figure 4:**
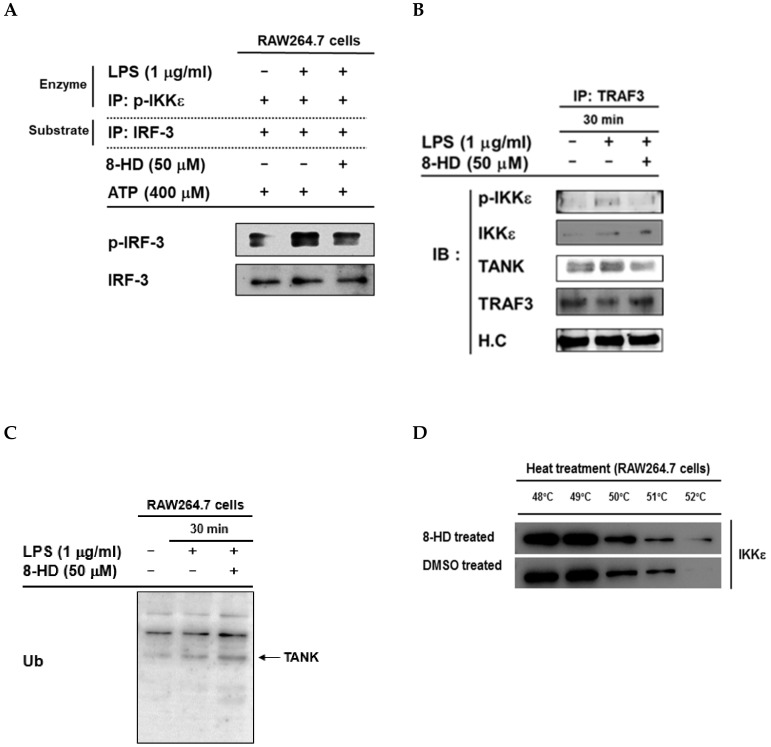
8-HD inhibits the kinase activity of IKKε and interferes with the TRIF-mediated complex formation composed of TRAF3, TANK, and IKKε. (**A**) Immunoprecipitation and in-vitro kinase assay of p-IKKε as an enzyme and IRF-3 as a substrate. Immunoprecipitates of p-IKKε from LPS-induced RAW264.7 cells were incubated with immunoprecipitates of IRF-3 for 1 h in the presence of ATP (400 μM) with or without 8-HD (50 μM) as indicated. Phospho- and total levels of IRF-3 were then determined by immunoblotting. (**B**) The effect of 8-HD on the TRAF3 and IKKε interaction. Endogenous TRAF3 was immunoprecipitated from LPS-induced RAW264.7 cells pre-treated with or without 8-HD (50 μM). The level of phosphorylated IKKε was determined by immunoblotting. H.C: Heavy chain. (**C**) Treatment of 8-HD has a marginal effect on polyubiquitination. RAW264.7 cells (5 × 10^6^ cells/mL) were pre-incubated with MG132 (50 mM) for 4 h. The cells were then pre-treated with 8-HD (50 μM) for 30 min, followed by incubation with LPS (1 μg/mL) for 30 min. The level of polyubiquitination was analyzed by immunoblotting of whole cell lysates with ubiquitin (Ub) antibody. (**D**) RAW264.7 cells (2.5 × 10^7^ cell/mL) were grown overnight followed by 8-HD (50 μM) or DMSO treatment for 30 min and LPS (1 μg/mL) for 30 min afterward. CETSA was performed and the remaining soluble protein of IKKε was analyzed by immunoblotting analysis.

**Figure 5:**
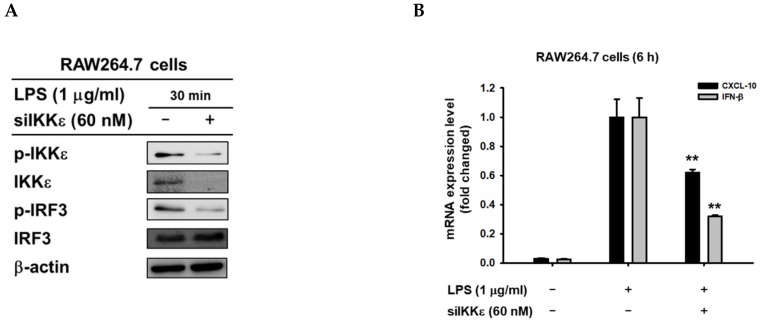
Knockdown of IKKε negatively regulates IRF3 phosphorylation and the expression of IRF3-dependent genes. RAW264.7 cells were transfected with siRNA (60 nM) targeting to IKKε for 48 h followed by treatment of LPS (1 μg/mL) for indicated times. (**A**) Phospho- and total levels of IKKε and IRF-3 were then determined by immunoblotting. (**B**) The mRNA analysis of CXCL-10 and IFN-β was performed using real-time PCR. ∗∗ *p* < 0.01 compared to the control group (LPS alone).

Based on previous results, IKKε tends to be the target enzyme inhibited by 8-HD. Here we employed an in-vitro kinase assay to assess whether 8-HD can directly inhibit phosphorylation of IRF-3 by IKKε. We performed immunoprecipitation of endogenous p-IKKε from LPS-stimulated RAW264.7 cells, used as an enzyme for the in-vitro kinase assay. In parallel, we also immunoprecipitated endogenous IRF-3 from RAW264.7 cells, used as a substrate for the in-vitro kinase assay. The in-vitro kinase assay was performed by incubating the enzyme and substrate in the presence of ATP, as well as in the presence or absence of 8-HD (50 μM) as indicated. The reaction was terminated after 1 h of incubation by adding 1 × sample buffer and was then immediately analyzed by immunoblotting. According to the immunoblotting result, phosphorylation of IRF-3 was found to be increased in LPS-stimulated RAW264.7 cells, while 8-HD treatment showed a decrease in IRF-3 phosphorylation (Figure 4A). This result implies that 8-HD was able to directly inhibit the kinase activity of IKKε to phosphorylate IRF-3. In addition, when we overexpressed IKKε, we also observed the autophosphorylation of IKKε, whereas 8-HD treatment did not show any effects (Figure S1C), suggesting that inhibitory action of 8-HD can be seen at the substrate phosphorylation level, but not autophosphorylation activity of IKKε. To highlight the importance of IKKε role in the IRF-3 signaling pathway, we re-evaluate it by performing depletion of IKKε. Depletion of IKKε indicates the downregulation of IRF-3 target genes expression (Figure 5A) and inhibition of IRF-3 activation (Figure 5B).

To show that 8-HD indeed can regulate IKKε, we performed immunoprecipitation of TRAF3 following immunoblotting of p-IKKε to determine whether 8-HD regulates a complex formation between TRAF3 and IKKε in LPS-stimulated RAW264.7 cells. The immunoblotting results clearly indicated that pre-treatment with 8-HD decreased the level of p-IKKε but not its total form in the complex state with TRAF3 (Figure 4B). TANK was also found to be decreased in the binding complex composed of p-IKKε and TRAF3 during 8-HD treatment. In addition, we tried to analyze the effect of 8-HD on polyubiquitination activity of whole proteins after LPS induction. The results showed that there was a clear increase in the polyubiquitination of proteins in the 8-HD/LPS-treated group compared to LPS alone at 30 min (Figure 4C), implying that ubiquitination might be a critical phenomenon to decide the binding levels of TRAF3 and p-IKKε. To confirm that 8-HD can indeed interfere with complex binding, we tried to confirm the engagement of 8-HD in the complex protein of IKKε and TRAF3. CETSA was performed using various heating temperatures based on a previous report [31]. According to the immunoblotting results of IKKε (Figure 4D), we can have observed the shifted of IKKε protein level upon 8-HD treatment compared with DMSO-treated condition. This shifted pattern indicated the thermostabilization of IKKε as a result of engagement with 8-HD, which strengthens our view that 8-HD can interfere with the complex formation between IKKε and TRAF3.

**Figure 6:**
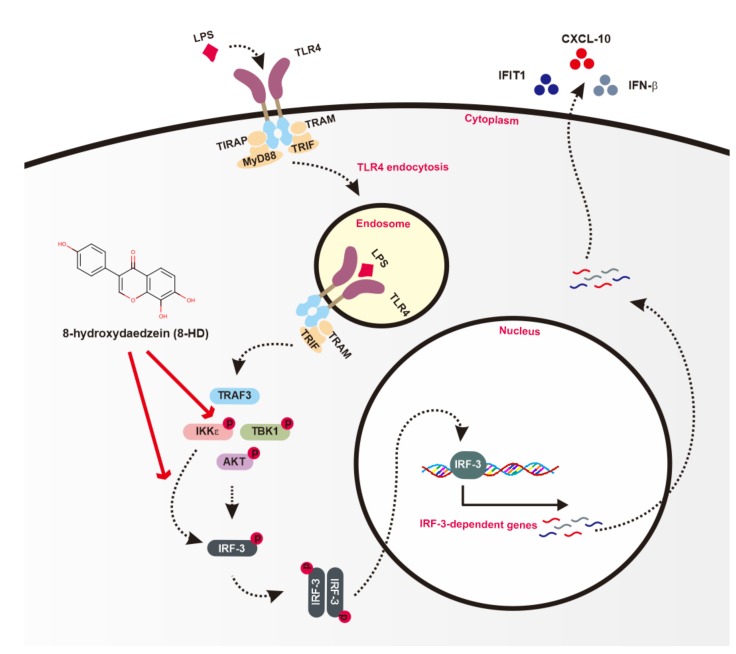
Putative inhibitory effect of 8-HD in the IRF-3 signaling pathway. 8-HD inhibits IRF-3 activation and subsequently IRF-3-dependent gene transcription by direct suppression of kinase activity of IKKε and by interfering with complex formation composed of TRAF3, TANK, and IKKε.

Taken together, these results imply that 8-HD was able to directly inhibit the kinase activity of IKKε to induce phosphorylation of IRF-3. In addition, 8-HD was also able to decrease molecular interaction between TRAF3, TANK, and IKKε upon TRIF activation conditions thereby reducing the activation of AKT and IRF-3 (Figure 6).

## 4. Discussion

Increasing evidence has highlighted the potential pharmacological benefits of 8-HD, including anti-inflammatory activities [13,15,16]. Although a few of these reports have described some inhibitory mechanisms by which this compound exhibits its anti-inflammatory activities, the exact mechanism remains to be determined. Particularly, there has been no report regarding the regulatory role of this compound upon the IRF-3 signaling pathway in inflammation. Undeniably, considering the importance of the IRF-3 pathway in inflammation-related diseases such as rheumatoid arthritis, systemic lupus erythematosus, systemic sclerosis, inflammatory bowel disease, chronic obstructive pulmonary disease, and type II diabetes in addition to chronic hepatitis [22], we determined whether 8-HD could also regulate this pathway. In the present study, we explored the anti-inflammatory mechanism of 8-HD with respect to the IRF-3 signaling pathway using LPS- and poly (I:C)-stimulated murine macrophages as an in-vitro model of inflammation. 

PCR results indicated 8-HD downregulated mRNA expression of these IRF-3-dependent genes in a dose-dependent manner. This result supports our hypothesis that 8-HD regulates the IRF-3 signaling pathway. It should be noted that the molecular mechanism leading to activation of IRF-3 remains elusive, although some of the regulatory enzymes have been identified and established [22]. Relating to our context, binding of LPS as a ligand to TLR-4 mediates activation of the signaling cascade in a MyD88-independent manner by utilizing adaptor proteins TRIF and TRAM [32]. In addition, a synthetic dsRNA mimic Poly I:C can be recognized by TLR-3 and also promotes signaling activation in a TRIF-dependent manner. Both of these signaling cascades lead to the formation of TRAF3/TANK/TBK1/IKKε complex, which facilitates kinase activation of TBK1 and IKKε to phosphorylate and activate IRF-3 [33,34,35,36]. AKT, a PI3K downstream enzyme, had recently been studied in IRF-3 signaling due to its ability to interact with TBK1. Both TBK1 and IKKε can directly phosphorylate AKT at Thr-308 and Ser-473 and are required for complete activation of IRF-3 [19,28,29,37]. A hallmark of IRF-3 activation is a phosphorylation event of IRF-3 at Ser396 that mediates this protein to undergo conformational changes allowing dimer formation and subsequent translocation into the nucleus to promote IRF-3-dependent gene transcription [22,38].

Our finding indicated that 8-HD was able to inhibit IRF-3 activation, shown by decreased phosphorylation level of IRF-3 at Ser396 (Figure 3A) and nuclear translocation of IRF3 (Figure S2). Analysis of upstream enzymes of IRF-3 demonstrated that 8-HD was able to decrease the phosphorylation level of AKT. This result is in agreement with a previous report that 8-HD can inhibit AKT phosphorylation [15]. Furthermore, we also found that 8-HD treatment decreased the phosphorylation level of IKKε but had no effect on phosphorylation of TBK1. To clarify these effects, we performed overexpression of TBK1-WT, TBK1-ΔCC, and AKT isoforms (AKT1 and AKT2) in HEK293T cells. As shown in Figure S1A, overexpression of both TBK1-WT and TBK1-ΔCC induced autophosphorylation of TBK1 in HEK293T cells, which is in agreement with a previous report [30]. As expected, 8-HD treatment had no effect on the autophosphorylation of TBK1. Moreover, when we overexpressed AKT under similar conditions, we observed no alteration in the autophosphorylation level of AKT in the 8-HD treatment group (Figure S1B). This result implied that the downregulation of AKT phosphorylation by 8-HD, as observed in Figure 3B, might be due to the inhibition of upstream enzymes rather than direct inhibition of AKT kinase activity. This suggests that IKKε tends to be the target inhibitory enzyme which is regulated by 8-HD. 

To confirm our hypothesis, we performed an in-vitro kinase assay using immunoprecipitated p-IKKε from LPS-stimulated RAW264.7 cells as an enzyme source and immunoprecipitated IRF-3 as a substrate source from untreated RAW264.7 cells. This assay was conducted to determine whether 8-HD can directly inhibit the phosphorylation of IRF-3 by activated IKKε. The immunoblotting result indicated that 8-HD can directly inhibit phosphorylation of IRF-3 triggered by activated IKKε (Figure 4A). Our results also indicate that 8-HD can act as an inhibitor to disrupt the interaction between TRAF3 and p-IKKε. The decreased phosphorylated level of IKKε then resulted in decreased phosphorylation of AKT, which is a downstream enzyme, to subsequently decrease IRF-3 activation. In addition, our finding also indicated that 8-HD was also able to destabilize TANK in the TRAF3-mediated complex assembly. Upon TLR4 activation, TRIF recruits a ubiquitination complex composed of TRAF3, UB2N, and UB2V2 (E2-enzyme) which, in turn, binds to the adaptor TANK coupled to IKKε [36]. It is possible that 8-HD might have acted to disrupt the interaction between TANK and p-IKKε in the TRAF3-mediated complex, resulting in suppression of IRF3 activation and subsequent induction of IRF3-mediated gene expression. To clarify that 8-HD engagement in the complex of IKKε and TRAF3, the CETSA was performed. The results suggested that upon heat treatment, 8-HD was able to make thermostabilization of IKKε which can be observed by the shifted protein level upon increasing heat treatment (Figure 4D). These results indicated that 8-HD was able to engage to the IKKε protein which then interferes with the binding complex with TRAF3 upon IRF-3 signaling activation.

So far, the exact mechanism as to how this compound can reduce the binding of TANK to this complex is not fully understood. Since stimulation of TANK degradation or blockade of protein-protein interaction between TANK, TRAF3, and p-IKKε are generally speculated pathway, whether 8-HD is able to modulate the activity of degradation or modification enzymes of TANK was simply examined using an antibody to ubiquitin. One of the interesting points that we have is the enhanced protein level of TRAF3 at 90 and 120 min upon 8-HD treatment conditions (Figure 3B), implying that degradation or destabilization of TRAF3 is not a major concern in 8-HD-mediated suppression of complex formation. Rather, Figure 4B strongly indicates that TANK might be a critical protein that can affect the binding levels of p-IKKε during 8-HD treatment. Unfortunately, however, current data are not enough for us to explain in detail the molecular mechanism as to how 8-HD decreased the binding level of the TANK. The previous understanding was that LPS-mediated TLR4 activation leads to the upregulation of polyubiquitination, which then stimulates the degradation of signaling proteins such as TRAF3 and TANK [33,39]. Therefore, it is considered that the increase in ubiquitination level during 8-HD treatment conditions (Figure 4C) might directly contribute to the downregulation of the TANK level in the signaling complex composed of p-IKKε, TRAF3, and TANK. Indeed, since the polyubiquitination level of proteins including TANK was apparently increased in 8-HD-treated cells (Figure 4C) at 30 min, we consider a possibility that suppression of TRAF3 binding to p-IKKε by 8-HD might be due to the induction of TANK degradation via enhanced polyubiquitination. Therefore, in terms of TNAK degradation, potential 8-HD-target degradation-inducing enzymes will be further examined in detail.

Moreover, the essential roles of IKKε in the regulation of IRF-3 signaling pathways are confirmed by the knockdown strategy of IKKε (Figure 5). Thus, the treatment of siRNA to IKKε showed to decrease IRF-3 phosphorylation and expression of IRF-3-dependent genes such as CXCL-10 and IFN-β in response to LPS (Figure 5B). 

## 5. Conclusions

The present study aimed to clarify the anti-inflammatory activity of 8-HD and to identify the molecular target of inhibition through the IRF-3 signaling pathway. Here, 8-HD specifically acts as an inhibitor to block the kinase activity of IKKε and to decrease molecular interaction between TRAF3 and IKKε, following downregulation of IKKε and Akt phosphorylation thereby inhibiting IRF-3 activation and finally reducing the expression of IRF-3-dependent genes such as *IFN-β*, *CXCL10,* and *IFIT1* (Figure 6). In addition, since we found 8-HD activity in regulation of IRF-3 signaling pathway with general in vitro model of inflammation, the related confirmation of its activity upon IRF-3-associated inflammatory disease condition such as atopic dermatitis, chronic hepatitis, rheumatoid arthritis, systemic lupus erythematosus, systemic sclerosis, inflammatory bowel disease, chronic obstructive pulmonary disease, and type II diabetes must be included in the future studies, including using in vivo model. These findings verify the potential activities of 8-HD for development as an anti-inflammatory agent that can inhibit multiple inflammatory transcription factors of NF-κB, AP-1, and IRF-3.

## Figures and Tables

**Table 1:** Primer sequences used for PCR.

PCR Type	Genes Name	Sequence (5′-3′)	
Semiquantitative RT-PCR	*IFN-β*	Forward	TCCAAGAAAGGACGAACATT
		Reverse	TGAGGACATCTCCCACGTCA
	*IFIT1*	Forward	ATGCAGTCGTAGCCTATCGC
		Reverse	CCTGCAAGGCCCTGTTTAGA
	*GAPDH*	Forward	ACCACAGTCCATGCCATCAC
		Reverse	CCACCACCCTGTTGCTGTAG
qPCR	*IFN-β*	Forward	AAGAGTTACACTGCCTTTGCTATC
		Reverse	CACTGTCTGCTGGTGGAGTTCATC
	*IKKε*	Forward	CCACTTGGAGTGCAGGAAGA
		Reverse	CCGGAT TTCTTGTTTCGGGC
	*CXCL10*	Forward	ATCATCCCTGCGAGCCTATCC
		Reverse	CGGATTCAGACATCTCTGCTCATC
	*IFIT1*	Forward	ATGCAGTCGTAGCCTATCGC
		Reverse	CCTGCAAGGCCCTGTTTAGA
	*GAPDH*	Forward	CAATGAATACGGCTACAGCA
		Reverse	AGGGAGATGCTCAGTGTTGG

## Supplementary Materials

The following are available online at https://www.mdpi.com/2218-273X/10/2/238/s1, Figure S1: 8-HD does not affect autophosphorylation of TBK1, AKT, and IKKε. Figure S2: 8-HD inhibits nuclear translocation of IRF3. Figure S3: Replicates results of immunoprecipitation experiments.

## Abbreviations

8-HD	8-hydroxydaidzein
NF-κB	nuclear factor-κB
AP-1	activator protein-1
IRF-3	interferon regulatory factor -3
LPS	lipopolysaccharide
AKT	protein kinase B
TRAF3	TNF receptor associated factor 3
IFN-β	Interferon-β
CXCL10	(C-X-C motif) chemokine 10
PAMPs	pathogen-associated molecular patterns
PRRs	pattern recognition receptors
TLRs	Toll-like receptors
RLRs	RIG-I-like receptors
cGAS	cyclic GMP-AMP synthase
STING	stimulators of interferon genes
CCL5	(C-C motif) ligand 5
Nrf2	Nuclear factor erythroid 2–related factor 2
TAK1	Transforming growth factor beta-activated kinase 1
MTT	3-(4,5-dimethylthiazol-2-yl)-2,5-diphenyltetrazolium bromide
IKKε	inhibitor of nuclear factor kappa-B kinase subunit epsilon
TBK1	TANK binding kinase 1
ATP	Adenosine 5′-triphosphate
TRIF	TIR-domain-containing adapter-inducing interferon-β
TRAM	TRIF–related adaptor molecule
MyD88	Myeloid differentiation primary response 88
PI3K	Phosphatidylinositol-4,5-bisphosphate 3-kinase
