# Supplementary material for: Regulation of 8-Hydroxydaidzein in IRF3-Mediated Gene Expression in LPS-Stimulated Murine Macrophages
Source: Biomolecules. 2020 Feb 4;10(2):238. doi: 10.3390/biom10020238 (PMC7072285; doi:10.3390/biom10020238)
Supplement: Supplementary file 1 [file biomolecules-10-00238-s001.pdf]

Supplementary Figures

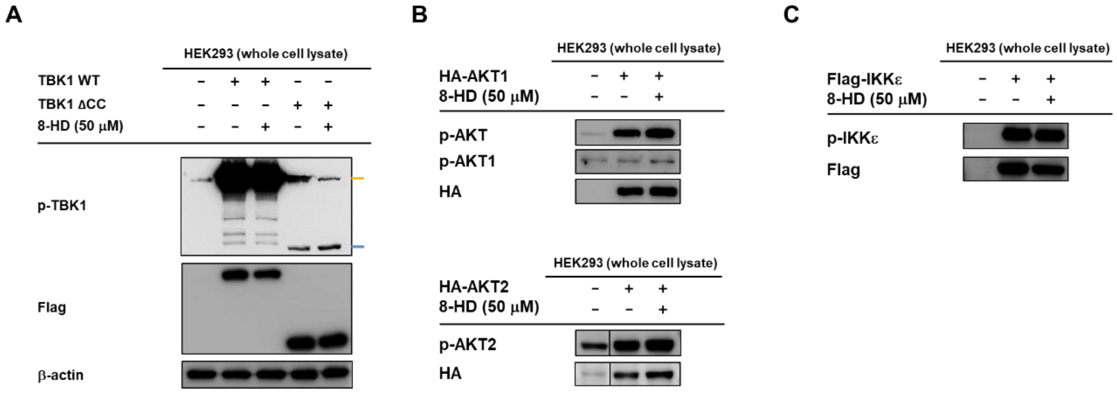

Figure S1. 8-HD does not affect autophosphorylation of TBK1 and AKT. HEK293T cells (10^6 cells/ml) were transfected using TBK1 WT, TBK1 ΔCC, HA-AKT1, HA-AKT2, or Flag-IKKε (0.8 μg/ml) as indicated for 24 h, followed by treatment with 8-HD (50 μM) or vehicle as indicated for additional 24 h without changing the media. (A) The effect of 8-HD on autophosphorylation of TBK1 was analyzed. The phosphorylation level of TBK1 and the level of Flag in whole cell lysates were analyzed by immunoblotting. (B) The effect of 8-HD on autophosphorylation of AKT was determined. The phosphorylation of AKT1, AKT2, and HA in whole cell lysates was analyzed by immunoblotting. (C) The effect of 8-HD on autophosphorylation of IKKε was determined. The phosphorylation of IKKε and Flag in whole cell lysates was analyzed by immunoblotting.

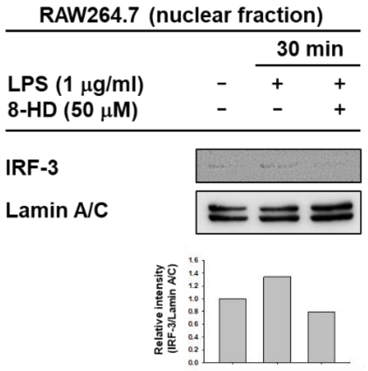

Figure S2. 8-HD inhibits nuclear translocation of IRF3. RAW264.7 cells (5 × 10^6 cells/ml) were pre-treated with 8-HD (50 μM) for 30 min, followed by incubation with LPS (1 μg/ml) for 30 min. The level of translocated IRF-3 was determined by immunoblotting of the nuclear fraction. Lamin A/C was used as a loading control for the nuclear fraction.

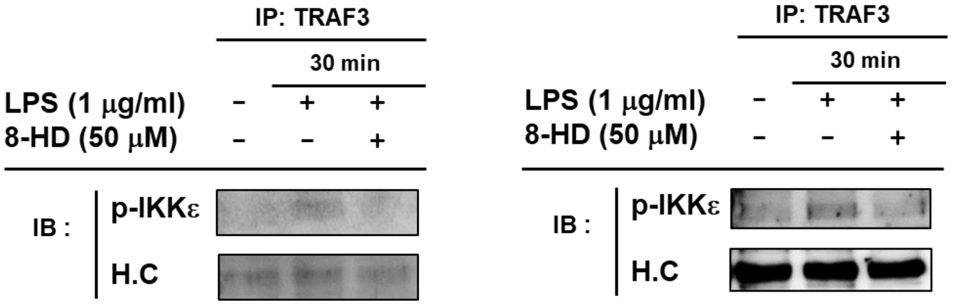

Figure S3. Replicates results of immunoprecipitation experiments. Endogenous TRAF3 was immunoprecipitated from LPS-induced RAW264.7 cells pre-treated with or without 8-HD (50 μM). The level of phosphorylated IKKε were determined by immunoblotting. H.C: Heavy chain.
